# Foamed Phase Change Materials Based on Recycled Polyethylene/Paraffin Wax Blends

**DOI:** 10.3390/polym13121987

**Published:** 2021-06-17

**Authors:** Patrik Sobolčiak, Miroslav Mrlik, Anton Popelka, Antonín Minařík, Marketa Ilcikova, Peter Srnec, Zuzana Nogellova, Mabrouk Ouederni, Igor Krupa

**Affiliations:** 1 Center for Advanced Materials, Qatar University, Doha 2713, Qatar; patrik@qu.edu.qa (P.S.); anton.popelka@qu.edu.qa (A.P.); 2 Centre of Polymer Systems, University Institute, Tomas Bata University in Zlin, Nad Ovcirnou 3685, 760 01 Zlin, Czech Republic; minarik@utb.cz (A.M.); ilcikova@utb.cz (M.I.); srnec@utb.cz (P.S.); 3 Department of Physic and Materials Engineering, Faculty of Technology, Tomas Bata University in Zlin, Nad Ovcirnou 3685, 760 01 Zlin, Czech Republic; 4 Polymer Institute SAS, Dúbravská cesta 9, 845 41 Bratislava, Slovakia; zuzana.nogellova@savba.sk; 5 QAPCO R&D–Qatar Petrochemical Company, Doha 756, Qatar; m.ouederni@qapco.com.qa

**Keywords:** phase change materials, foams, paraffin wax, recycled polyethylene, micro-computed tomography, dynamic mechanical analysis

## Abstract

Foamed phase-change materials (FPCMs) were prepared using recycled linear low-density polyethylene (LLDPE) blended with 30 wt.% of paraffin wax (PW) and foamed by 1,1′-azobiscarbamide. The protection of pores’ collapse during foaming process was insured through chemical cross-linking by organic peroxide prior foaming. This work represents one of very few attempts for a preparation of polymeric phase change foams without a use of micro-encapsulated phase change component leading to the enhancement of the real PCM component (PW) within a final product. The porous structure of fabricated foams was analyzed using micro-computed tomography, and direct observation, and reconstruction of the internal structure was investigated. The porosity of FPCMs was about 85–87 vol.% and resulting thermal conductivity 0.054–0.086 W/m·K. Differential Scanning Calorimetry was used to determine the specific enthalpies of melting (22.4–25.1 J/g) what is the latent heat of materials utilized during a heat absorption. A stability of samples during 10 heating/cooling cycles was demonstrated. The phase change changes were also investigated using the dynamic mechanical analysis from 0° to 65 °C during the 10 cycles, and the mechanical stability of the system and phase-change transition were clearly confirmed, as proved by DSC. Leaching test revealed a long-term release of PW (around 7% of its original content) from samples which were long term stored at temperatures over PW melting point. This is the usual problem concerning polymer/wax blends. The most common, industrially feasible solution is a lamination of products, for instance by aluminum foils. Finally, the measurement of the heat flow simulating the real conditions shows that samples containing PW decrease the energy passing through the sample from 68.56 to 34.88 kJ·m^−2^. In this respect, FPCMs provide very effective double functionality, firstly common thermal insulators, and second, as the heat absorbers acting through melting of the PW and absorbing the excessive thermal energy during melting. This improves the heat protection of buildings and reduces temperature fluctuations within indoor spaces.

## 1. Introduction

It is estimated that roughly 30–40% of the world’s total energy produced from fossil fuels is consumed by the construction industry, what results in one-third of the world’s greenhouse gas emissions. In Qatar, 65% of produced electricity is consumed by cooling systems in all types of buildings [1]. From this reason, it is important to develop materials which effectively absorb and release an excess thermal energy to insure thermal indoor comfort, minimizing the need of electrical energy for heating in winter and cooling in summer. These materials can reduce variations in temperature of buildings when outside temperature changes over day. The reduction of the energy consumption in buildings can be insured by effective thermal insulation or a heat absorption. Polymeric foams, such as polyurethane, polyethylene, and polystyrene are the most commonly applied materials for insulation because of their low thermal conductivity, low density, acceptable mechanical properties, and an appropriate price [2,3].

Another approach to decreasing the energy consumption for heating/cooling is based on Phase Change Materials (PCMs). PCMs are materials which undergo phase transition (usually from solid to liquid phase and vice versa) isothermally at relatively low, specifically selected temperature, which is associated with an absorption and a release of high amount of energy, proportionally to their specific heat of melting and crystallization. Various materials can be used as PCM; the most usual ones are inorganic salts and their eutectics, fatty acids, polyethylneglycol, and PWs [4]. PW are very suitable PCM because of their favorable properties, like a high specific enthalpy of melting and crystallization, negligible super-cooling, stability, easy availability, and an appropriate price in comparison to other PCMs [5]. Among disadvantages belong relatively high flammability in comparison with inorganic salts. The specific melting enthalpy of PW is in the range from 180 to 230 kJ·kg^−1^. This fact results in a high energy storage density of PW due to a high latent heat which accomplishes the melting and crystallization of PW [6,7]. It is evident that after melting, PW can leach from the system in which they are incorporated. The simplest way to suppress this effect is to keep PW in closed tanks or containers; however, there are some disadvantages resulting from this concept [4,5]. On the other hand, PW can be also maintained in the stable forms by their encapsulation within a polymeric shell or by their blending with selected polymers [8,9,10,11,12,13]. After blending, polymeric matrices keep PW in a compact form and partly suppress leaching after PW melting, depending on the chemical and morphological similarity of PW and selected polymers, viscosity, and a presence of the fillers. These materials are called Shape Stabilized PCM. Polyethylene (PE) is the most commonly employed polymeric material used for mixing with PW due to their structural and chemical similarity [14,15,16], which enables and incorporate of a large amount of PW into PE matrix (up to 60 wt.%) because of the components’ compatibility.

In recent years, foamed PCMs (particularly PU foams) have been extensively investigate in order to combine advantages of thermal insulation and thermal energy storage. The preference of PU is because despite the higher price against polystyrene and polyethylene, polyurethane foams are frequently used for thermal insulation of buildings due to excellent thermal insulation properties and low flammability. It has been reported that an appropriate content of various PCMs in a combined product can enhance the thermal energy storage capacity [17,18,19,20,21,22,23,24,25].

The incorporation of active PCM component into the foams can be realized by techniques which are classified as indirect and direct ones. The indirect methods employ either different containers for a macro-encapsulation or polymeric shells materials for a micro-encapsulation to suppress leakage. The direct techniques for an incorporation of PCM components involve: (i) impregnation of polymeric structures (mostly PU) by liquid PCM and (ii) polymerization of polymers together with added PCM active component [26].

In general, foams are mostly prepared from polymers, carbon-based (carbon nanotubes, graphene) materials, inorganic (silica formed by sol-gel processes) materials, and metals (aluminum). Metallic foams (copper, nickel, and aluminum) are other structures used as a skeleton for impregnation by PWs. The main reason is an enhancement of thermal conductivity filled aluminum foam by PW is 5–9 times higher than that of pure paraffin [27].

However, it has to be distinguished whether foamy structures are used as a skeleton only which is fully filled by PCM component, and thus, it contains high concentration of PCM component showing high latent heat, or as a hybrid structure, where pores filled by air and by PCM are balanced in some proportion. It means that fully filled foams have much higher content of PCM and thus high heat absorption capacity (a melting enthalpy is of the order tens J/g); however, those final products are not foams. The real PCM foams should contain an appropriate ratio between PCM component (e.g., PW) and air voids fractions [17,23,28,29,30,31].

Sarier [17] modified PU foams by n-hexadecane and n-octadecane. It was found that PU foams consisting of n-octadecane with a weight portion of PCM to PU equals to 1 to 1.4 showed a good heat absorption performance, having the specific enthalpy of melting of 77.8 J/g.

Melamine foams (MA) are highly porous materials which can be easily impregnated by PCM components. Tetradecylamine (TDA) was used as a thermal energy storage medium and melamine foam was used as the container. The melamine covalently bonded multi-walled carbon nanotubes were added to improve the thermal conductivity of final structure. Materials showed a large melting enthalpy (183.7 J/g), improved thermal conductivity and excellent cycle stability. However, the final material is not foam—a foam just serves as a skeleton.

**Figure 1:**
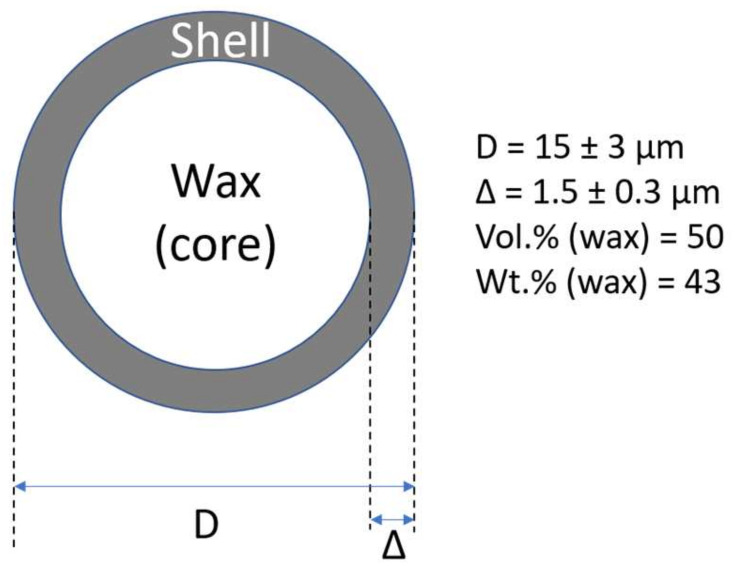
Typical shape and dimensions of synthesized microcapsules [33].

Polymeric foams can be also prepared from polymeric mixtures formed by thermoplastic matrix (polyethylene and encapsulated PCM using common procedures for a formation of thermoplastics; however, the shell content consumes a lot of space which is not usable for latent heat storage. It is evident from Figure 1, the PW (core content) in microcapsule has outer diameter of 15 ± 3 μm, and the thickness of the shell of 1.5 ± 0.3 μm is only 50 vol.% (43 wt.%). Therefore, even in highly filled composite structures close to the maximum packaging fraction (microcapsules content up to 60 vol.%), PW content is 30 vol.% [32,33].

In this paper, the preparation of foamed PCMs based on recycled linear low-density polyethylene and PW blends is reported using foaming 1′-azobiscarbamide and (2,5-dimethyl-2,5-di-(tert-butylperoxy)hexyne-3 as a cross-linking agent. The crosslinking by organic peroxide was performed prior the foaming to suppress the collapse of the developing pores.

The heat storage capacity given by the melting enthalpy is relatively low (around 20 J/g); however, it is comparable with values reported in literature for usual PU foams modified by various PCMs. PU foams modified by n-hexadecane, n-hexaoctane, and myristyl myristate have specific enthalpies of melting in the range from 4 to 77 J/g, mostly ranging from 15 to 20 J/g. Materials showing higher values show very low porosity, and the foam is used only as a skeleton for an impregnation. It means that the volume portion of unfilled voids (pores) is very low. Our results fall in the usual range of the specific melting enthalpies reported in literature [28,29].

It is worth to mention that recycled polyethylene was used in this work. The recycling or reuse of plastics in general and PE, particularly LDPE and LLDPE grades for packaging, is a topic of high priority today. The reuse of LDPE is currently very low due to limited possibilities to design any useful product with it. Simply said, packaging grades are suitable only for packaging, and after their use, they are not suitable for recycling for the same application due to many reasons such as complicated gathering and cleaning, deteriorated properties, a presence of additional components through packages’ post-treatment, etc. The utilization of recycled PE as a part of PCMs can be one of the few reasonable applications because some of the previously mentioned drawbacks are not important in this application.

## 2. Materials and Methods

**Figure 2:**
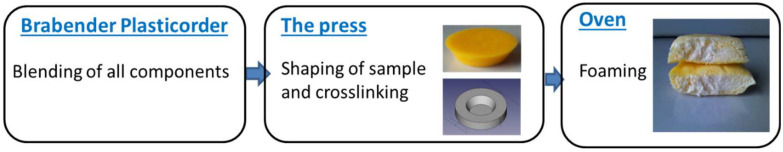
FPCM preparation.

Foamed PCMs were fabricated using recycled LLDPE (Twyla, Doha, Qatar), PW (Rubitherm Technologies GmbH, Berlin, Germany), a blowing agent 1,1′-azobiscarbamide, (Hangsun Plastic additives Co. limited, China), and a cross-linking agent (2,5-dimethyl-2,5-di-(tert-butylperoxy)hexyne-3 (Luperox130; Arkema, Colombes, France). Firstly, all the components (LLDPE/PW = 70/30 *w*/*w*, 10 wt.% of blowing agent, and 0.5 or 1 wt.% of cross-linking agent) were blended for 11 min at 140 °C in a Plastograph (Brabender GmbH & Co. KG, Duisburg, Germany). Then, the blended compound was hot pressed at 150 °C for 3 min in a cylinder-shaped mold to obtain the required shape, followed by further heating at 150 °C for 13 min to ensure the cross-linking of the mixture. Finally, the sample was put into an oven and kept at 150 °C for 25 min to allow decomposition of the blowing agent which led to the formation of the foamy material. The preparation of FPCM is schematically depicted in Figure 2.

For a comparison, foams without PW were prepared under the same conditions. The compositions of mixtures are summarized in Table 1. The portions of masterbatch and Luperox130 were related to the 100 g of LLDPE/PW mixtures (PW1, PW05) or to LLDPE only (P1, P05). For example, the composition 70/30/10/1 means that LDPE/PW mixture having the composition 70/30 *w*/*w* was prepared and then 10 wt.% of masterbatch and 1 wt.% of Luperox130 related to that mixture was added.

### 2.1. Differential Scanning Calorimetry (DSC)

The DSC measurements were performed by a Perkin Elmer DSC 8500 (Acron, OH, USA) differential scanning calorimeter. For measuring, the specific heat capacity common three-step method using sapphire as a standard was employed. The sample was cooled to 18 °C at a rate of 2 °C/min and held at this temperature for 3 min, then specimen was heated to 60 °C and held at it for 3 min. Calibration was done to sapphire standard. Nitrogen gas was passed through the instrument at a flow rate of 40 mL/min. DSC software was used for calculation of cp values. All experiments were repeated at least three times, and average values are presented.

In case of heating/cooling measurement cycling, temperature interval from 0 to 60 °C/min at rate 10 °C/min was employed.

The specific enthalpy of melting and crystallization, melting, and crystallization temperatures were determined from the second run.

### 2.2. Thermal Conductivity

The thermal conductivity was determined by a Hot Disk thermal analyzer (Hot Disk 2500, Göteborg, Sweden) using the transient place source (TPS) method [32]. A disk-shaped TPS sensor with diameter 3 mm was placed between two circular sample pieces. The measurement power ranged from 5 to 15 mW and the time of the measurement varied between 10 and 40 s. At least three measurements for each sample were performed at 25 °C with an accuracy within 3%.

### 2.3. Internal Foam Structure Investigation

The porosity of samples was investigated at the cut disk (dimensions) using computer micro-tomography (CT) on the SkyScan Unit (model 1174, Bruker, New York, NY, USA). Device was equipped with an X-ray power source (20–50 kV and maximum power 40 W) and X-ray detector (Bruker, New York, NY, USA). The CCD 1.3 Mpix unit was coupled to a scintillator by a lens with 1:6 zoom range. Projection images were taken at angular increment of 0.3° at a tube voltage of 30–40 kV and current of 585–730 μA. Duration of exposure was set to 15–30 s without the use of filter. 3D reconstructions were created via pre-installed CT image analysis software (v1.16.4.1, Bruker, New York, NY, USA). Porosity and corresponding standard deviation are calculated from three individual cylindrical sections.

### 2.4. Mechanical Investigation during Phase Change Cycling

In order to investigate the mechanical properties and their sustainability during phase change transition, dynamic mechanical analyzer (DMA 1, Mettler Toledo, Columbus, OH, USA) was used. The measurements were performed at 1 Hz in linear viscoelastic region, when the deformation was set to 1%. The temperature sweeps from 10 to 65 °C in 10 cycles were measured, and storage modulus and tan delta as a crucial parameter were elucidated and discussed.

### 2.5. Capability of the Energy Transfer

The heat flux from the source of 45 °C was measured using (GreenTEG, Rümlang, Switzerland, Zürich) and energy transfer through the foams was calculated using Equation (1):

(1) $$E=\int Wdt\times \frac{15}{45-T_{S}}$$

where *E* is energy passed through the material, *W* is heat flux at investigated time period, and *Ts* is surface temperature of the material before measurement.

## 3. Results

### 3.1. Internal Foam Structures Investigation

**Figure 3:**
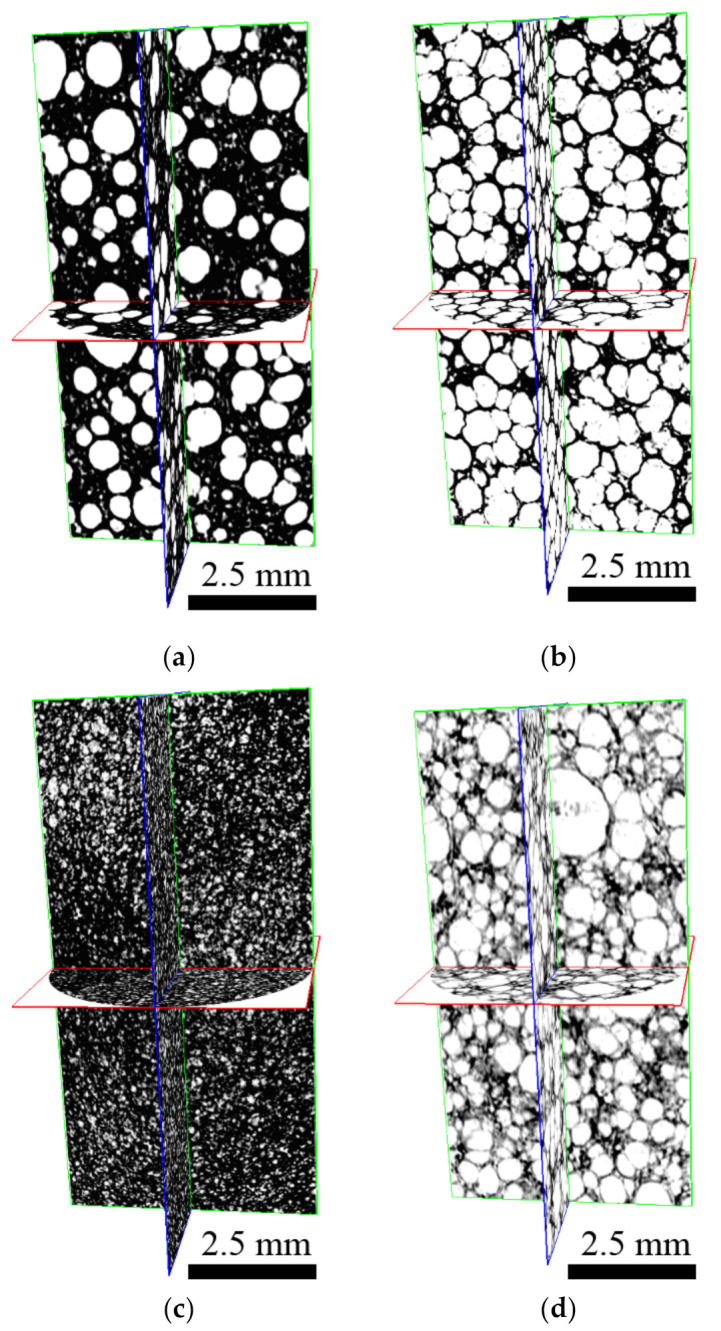
Space cross section of the foam sample: (**a**) PW1; (**b**) PW05; (**c**) P1; (**d**) P05.

As can be seen from Figure 3 and Table 2, an overall amount of the closed pores, their size, and homogenous distribution strongly depend on the amount of the cross-linker as well as on the presence of the PW in the system, as was already investigated by other authors [34,35]. With increasing amount of cross-linker porosity significantly increasing. In Figure 3a–d, real structures of the foams are presented, since the samples were not deformed during the cutting, and thus, the whole 3D structure is presented. All samples exhibit very similar porous structure, with exception of the sample P1. The sample P1 with 1% of cross-linker without PW showed a significantly different structure, most probably due to different viscosity (higher) during foaming step. On the other hand, the amount of cross-linker has impact on the number of closed pores and the higher the content of cross-linker is provided, the higher the number of closed pores is received. Moreover, the presence of PW decreases the number of pores; this is in close connection with the viscosity during the foaming step, and where the low the viscosity of the melt is reached, the larger pores are obtained [36]. It has to be mentioned here, that the approach of the computation tomography utilization applied in this article is very rare in the field of phase change materials of the polymer/graphite composites and PW; therefore, the comparison of the results with other literature sources is rather limited.

The volume portion of the pores was calculated from Equation (2):

(2) $$\varphi_{pores}\left(\%\right)=1-\frac{\rho_{foamed}}{\rho_{unfoamed}}\times 100\%$$

where *φ_pores_* is the volume portion of the pores and *ρ_foamed/_ρ_unfoamed_* are the specific densities of the unfoamed and foamed materials. The pore sizes and the gel content of cross-linked materials are listed in Table 3. It is evident that the gel portion of cross-linked LLDPE is higher than the gel portion of the LLDPE/PW blend. This is due to a proportional distribution of cross-linker within both the PW and LLDPE and W. This leads to the lowering of an effective concentration of peroxide in LLDPE, and thus, the crosslinking efficiency is lower. Crosslinking of low molecular weight PW is not possible at such low crosslinker content due to its short chains, and therefore, it does not contribute to the final gel content. The gel content is directly related to the crosslinking density of materials.

In order to suppress the collapse of the pores, materials were crosslinked prior to blowing. The volume portion of the pores within a foamed material was about 69 vol.%, which is a significantly lower value in comparison to the volume portion of the pores in original, neat LLDPE, which is about 92%. It indicates that only LLDPE phase is foamed, because PW component is not cross-linked, and thus, it does not have a sufficient viscosity to preserve pores.

### 3.2. Thermophysical Properties

The thermal conductivities of foamed samples are summarized in Table 4. Firstly, unfoamed PE had a thermal conductivity of 0.38 W/m·K, and the addition of PW caused its decrease to 0.33 W/m·K due to the lower thermal conductivity of PW, which is approximately 0.2 W/m·K. Secondly, all the foamed materials have significantly lower thermal conductivity than neat polyethylene and its blends with PW. This is caused by high porosity of materials in all cases due to very low thermal conductivity of air or gases in general. Higher porosity results in lower thermal conductivity. The crosslinking is another effect influencing thermal conductivity of semicrystalline materials, such are polyethylenes and PWs because it reduces their degree of crystallinity. Crystalline phase has almost six times higher thermal conductivity than the amorphous one. Eiermann [37] determined thermal conductivities of amorphous and crystalline phase of polyethylene of 0.091 and 0.593 W/K, respectively. Thus, higher crosslinking efficiency and higher porosity leads to the decreasing in thermal conductivity of final system; however, crosslinking efficiency also influences a formation of stabile pores, so these two phenomena are closely interconnected. As a consequence of various parameters influencing the final thermal conductivity, the lowest value of 0.066 W/m·K was determined for P05 sample.

DSC analysis of PW1, PW05, P1, and P05 is summarized in Table 5. Samples PW1 and PW05 contain LLDPE and paraffin. PW melting/crystallization temperature was around 42 or 38 °C, respectively, and the enthalpies ranged from 22.4 to 24.7 J/g. LLDPE exhibited melting temperature at 117 °C for PW1 and 121.6 °C for PW05 with enthalpies around 81 J/g.

**Figure 4:**
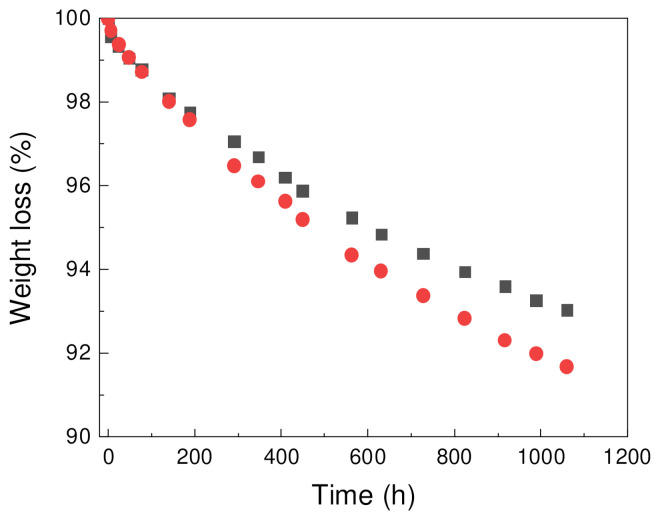
Weight loss of PW1 (squares) and PW05 (circles) samples over time.

Weight loss of foams was studied for samples contain paraffin, PW1 and PW05 (Figure 4). Both samples exhibited loss weight over time with achieved weight loss up to 8 wt.% of materials for PW05 sample. For PW1 sample, 7 wt.% weight loss was observed. Material which was lost during the leakage is expected to be predominantly PW as was previously reported for PCM samples with similar composition [38,39].

Table 6 shows comparison of PW1 and PW05 samples before and after leakage experiment focused on PW region. Solid-liquid and liquid-solid transition temperature was only slightly influenced by the leakage experiment. However, decreasing of both melting and crystallization enthalpies after leakage experiment has been observed proportionally to weight loss showed at Figure 4 indicating the leakage of PW.

**Figure 5:**
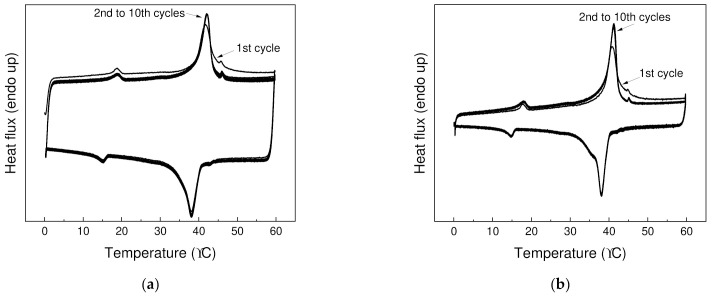
Thermal cycling for (**a**) PW1 and (**b**) PW05 at temperature from 0 to 70 °C over 10 heating and 10 cooling cycles.

The reproducibility of the storage and the release of thermal energy over longer period of time is crucial for applying PCMs in thermal management. Hence, thermal cycling (10 heating and 10 cooling cycles) from 0 to 60 °C was performed (Figure 5), and good reproducibility of storage and release of energy was observed. After first heating cycle, where thermal history of specimen is eliminated, almost identical thermographs have been observed for both PW1 and PW05.

### 3.3. DMA

**Figure 6:**
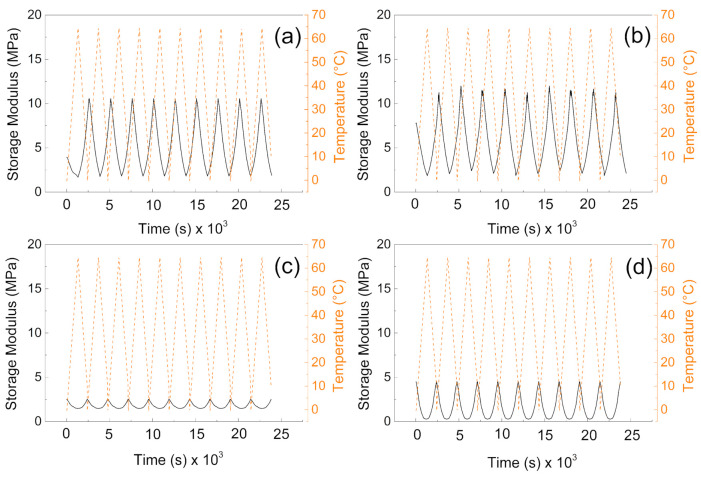
DMA investigation of the (**a**) P1, (**b**) P05, (**c**) PW1, and (**d**) PW05.

From the mechanical point of view, the samples without the PW show higher values of the storage moduli indicating better mechanical properties after compression, and more mechanical energy can be stored upon deformation. The values are very similar to those obtained by other authors [40]. Interestingly, the higher amount of the cross-linker does not provide significantly higher storage moduli, due to the fact the lower amount of the cross-linking agent provides pores with higher volume and thus contributes to the mechanical capability of the sample upon cyclic deformation over various temperature cycles. It can be seen in Figure 6a,b, that samples without PW can overcome only nine cycles of deformation while the temperature sweeps were 10 cycles. However, in both cases (with and without PW), the mechanical sustainability of the fabricated systems is stable over all 10 cycles performed. Therefore, the fabricated materials provide mechanical properties with storage moduli from 10 MPa to 15 MPa without PW, while the presence of the PW decreases the mechanical properties 5 times for 1% cross-linker and only 3 times for 0.5% cross-linker in the system.

**Figure 7:**
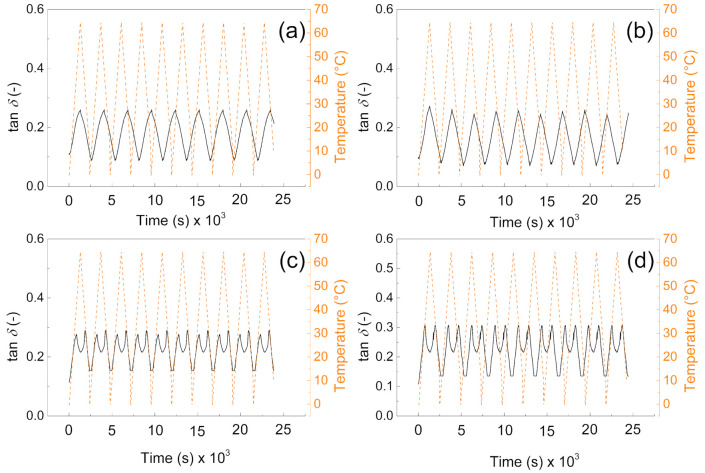
DMA investigation of the (**a**) P1, (**b**) P05, (**c**) PW1, and (**d**) PW05.

It can be clearly seen from Figure 7a,b that tan delta of the materials is very similar and reaches values from 0.1 to 0.3 depending on the applied temperature. Moreover, due to the absence of the PW, there is no response on the temperature change. However, in the case of samples (Figure 7c,d) there is visible phase change transition since the tan delta shows local maxima due to the PW melting. Beside this fact, they also not exceeded value 0.3 in all cases, and they are reversible in all 10 performed cycles; this behavior clearly indicates that from the mechanical point of view, the fabricated samples show excellent sustainability over time and temperature. The values of tan delta are around 0.3 indicating the elastic behavior with low internal heat energy dissipation during mechanical cycling. Our group in the former article obtained similar results regarding the dynamic mechanical response of such foams [41]; however, in the absence of the graphite particles, those in this amount just significantly influence the PW leaching capabilities as was investigated deeper in this paper.

**Figure 8:**
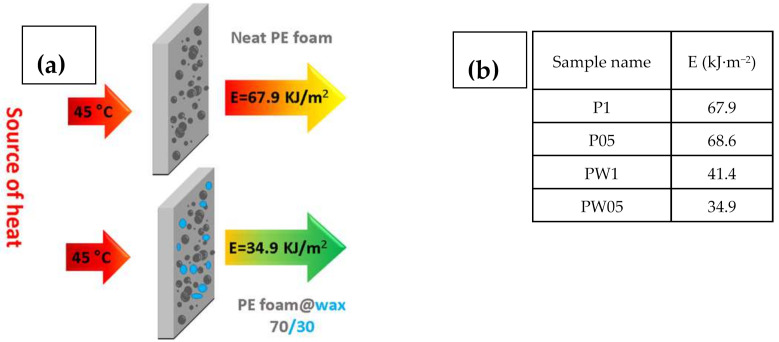
Schematic illustration describing the measurement principle (**a**) with calculated energies passed through the investigated samples (**b**).

To show, how the fabricated materials can be applied in the real-life applications, the heat flux investigations and calculated energies passing through the samples were calculated. Schematic illustration and table with calculated energies is depicted in Figure 8.

**Figure 9:**
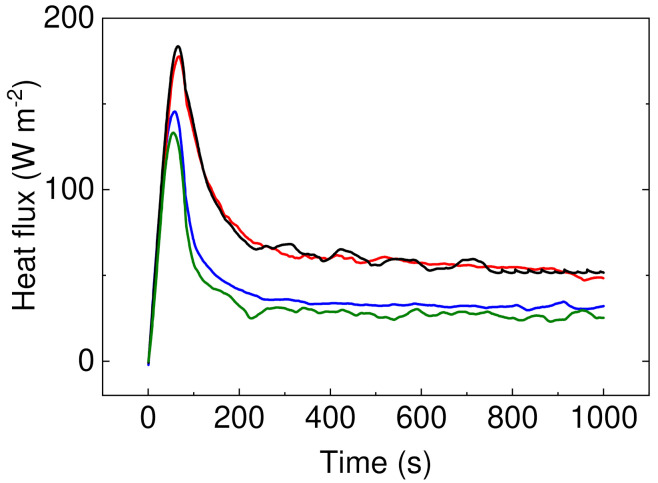
Heat flux development over the period of time upon 40 °C stimulation. Sample P05 is black line, P1 is red line, PW1 is blue line, and PW05 is olive line.

It can be seen that samples containing PW have significantly improved resistance against energy transfer, indicating that these samples do not allow nearly 50% of the energy in case of sample PW05 in comparison to sample P1. Moreover, the height of the peak is lower for the samples with PW (Figure 9), indicating that even though these samples show lower thermal conductivity, the considerable heat consumption of the PW plays a crucial role. In this respect, there is no visible peak of the PW phase change due to the fact that measurements were performed upon constant heating of 45 °C and checking the heat flow passing through the material and have different principle than that published by Sobolciak et al. [42]. A combination of the capability to significantly reduce the amount of the heat energy passing through the sample and further possibility to store this energy within the sample structure provide a promising approach to the passive heat energy storage systems with excellent mechanical properties.

## 4. Conclusions

This work focused on the preparation of foamed phase-change materials using recycled linear low-density polyethylene blended with 30 wt.% of PW. The blends were foamed by 1,1′-azobiscarbamide and cross-linked by organic peroxide (Luperox 130) to suppress the collapse of pores during foaming. The concentration of 30 wt.% of PW was selected according to previous optimization to get materials with appropriate latent heat (given by the specific enthalpy of melting), high porosity, low thermal conductivity, and acceptable leakage of PW from final materials.

The porous structure of fabricated foams was analyzed using micro-computed tomography and direct observation, and reconstruction of the internal structure was investigated. The amount of the closed pores was investigated, and it was shown that major impact lies on the amount of the cross-linker rather than PW. Overall porosity of FPCMs was about 85–87 vol.%, and resulting thermal conductivity was 0.054–0.086 W/m·K, which represents a significant decrease in thermal conductivity against pure PE (0.34 W/m·K). Differential Scanning Calorimetry was employed to determine the specific enthalpies of melting (22.4–25.1 J/g), which is the latent heat of materials utilized during a heat absorption. These results are in line with the results published in literature for foamy phase change materials. A reproducibility of the measurements during 10 heating/cooling cycles was demonstrated.

Leaching test revealed a long-term release of PW from samples, which were long term stored at temperatures over PW melting point. The amount of leached paraffin was about 7% of its original content after keeping samples at 60 °C for 1000 h. This is the usual problem concerning polymer/PW blends, and the most common, industrially feasible solution is a lamination, for instance by aluminum foils, as used by some producers such as Dupond.

The phase change capabilities were investigated also using the dynamic mechanical analysis from 0° to 65 °C during the 10 cycles, where the mechanical stability of the system and phase-change transition were clearly confirmed, and a decrease of the elastic modulus 5 times for samples without PW and 3 times with PW was presented. Moreover, the measurement of the heat flow simulated the real conditions and showed that samples containing PW decrease the energy passing through the sample from 68.56 to 34.88 kJ·m^−2^.

In this regard, newly developed foamy PCMs provide effective double functionality. First, they serve as standard thermal insulators, and second, the PW acts as a phase change component that absorbs thermal energy (the latent heat) during melting if the temperature increases above its melting point, which ensures better heat protection of buildings, for instance, against overheating and reduces temperature fluctuations within indoor spaces.

## Figures and Tables

**Table 1:** Composition of prepared foams.

Sample Name	LLDPE	PW	Blowing Agent	Luperox130
	wt.%	wt.%	wt.%	wt.%
PW1	70	30	10	1
PW05	70	30	10	0.5
P1	100	0	10	1
P05	100	0	10	0.5

**Table 2:** Results from 3D image analysis of foams.

Description	Abbreviation	Unit	Samples’ Codes			
			PW1	PW05	P1	P05
Total analyzed volume	TV	mm^3^	193	193	193	193
Foam volume in analyzed volume	vol. TV	mm^3^	120	69	162	83
Number of closed pores	No.	/	22,422	11,199	325,625	12,985

**Table 3:** The specific density, gel content, and volume portion of materials.

Sample	Density (g/cm^3^)	*φ_pores_* (vol.%)	Gel Portion (wt.%)
LLDPE	0.941	/	0
PW1	0.084	86.8	59.7 (0.1)
PW05	0.080	85.0	43.9 (0.8)
P1	0.143	83.6	84.1 (0.4)
P05	0.128	84.1	76.0 (1.0)

**Table 4:** Thermal properties of foams.

Sample	λ, W·m^−1^·K^−1^	k, mm^2^/s	c_p_, J·g^−1^·K^−1^
PW1	0.066	0.269	2.92
PW05	0.054	0.393	1.72
P1	0.086	0.459	1.31
P05	0.073	0.460	1.24

λ: thermal conductivity; k: thermal diffusivity; c_p:_ specific heat capacity.

**Table 5:** DSC characterization of foams. The standard deviations are shown in brackets.

	Heating				Cooling			
	Paraffin		LLDPE		Paraffin		LLDPE	
	T_m_ (°C)	ΔH_m_ (J/g)	T_m_ (°C)	ΔH_m_ (J/g)	T_c_ (°C)	ΔH_c_ (J/g)	T_c_ (°C)	ΔH_c_ (J/g)
PW1	42.7 (0.4)	22.4 (1.1)	117.2 (0.5)	81.1 (4.0)	38.4 (0.1)	23.3 (0.1)	110.2 (0.6)	82.7 (3.8)
PW05	42.9 (0.4)	25.1 (1.1)	121.6 (0.6)	80.8 (3.4)	37.1 (0.4)	24.7 (0.2)	112.4 (0.6)	80.9 (0.1)
P1	N/A	N/A	126.8 (0.4)	114.4 (2.7)	N/A	N/A	114.4 (1.1)	115.0 (1.6)
P05	N/A	N/A	128.2 (0.7)	113.1 (3.8)	N/A	N/A	116.5 (0.6)	116.8 (4.2)

T_m_: melting temperature; ΔH_m_: melting enthalpy; T_c_: crystallization temperature; ΔH_c_: crystallization enthalpy.

**Table 6:** DSC analysis of PW1 and PW05 foams before and after leakage.

Sample	Heating				Cooling			
	Ts-s (°C)	∆Hs-s (J/g)	Ts-l (°C)	∆Hs-l (J/g)	Ts-s (°C)	∆Hs-s (J/g)	Ts-l (°C)	∆Hs-l (J/g)
PW1	N/A	N/A	39.9	19.5	12.0	0.9	36.6	19.9
PW1afterL	16.1	0.9	39.7	14.8	12.4	0.8	36.3	15.0
PW05	N/A	N/A	41.4	20.6	N/A	N/A	36.7	19.7
PW05afterL	16.6	0.8	40.1	14.0	13.0	0.6	36.9	13.5

## Data Availability

Not applicable.
